# Anti-Obesity and Anti-Adipogenic Effects of Chitosan Oligosaccharide (GO2KA1) in SD Rats and in 3T3-L1 Preadipocytes Models

**DOI:** 10.3390/molecules26020331

**Published:** 2021-01-11

**Authors:** Jung-Yun Lee, Tae Yang Kim, Hanna Kang, Jungbae Oh, Joo Woong Park, Se-Chan Kim, Minjoo Kim, Emmanouil Apostolidis, Young-Cheul Kim, Young-In Kwon

**Affiliations:** 1 Department of Food and Nutrition, Hannam University, Daejeon 34054, Korea; seembeeks@hanmail.net (J.-Y.L.); xodid5606@naver.com (T.Y.K.); hanna9506@hanmail.net (H.K.); minjookim@hnu.kr (M.K.); 2 Institute of Functional Foods, Kunpoong Bio Co. Ltd., Jeju 63010, Korea; denisoh89@gmail.com; 3 Natural Products Institute, Biostream Technologies Co. Ltd., Gyeonggi-Do 17098, Korea; pjwrnds@biostream.co.kr; 4 Department of Bio Quality Control, Korea Bio Polytechnic, Chungnam 32943, Korea; sechage@kopo.ac.kr; 5 Department of Chemistry and Food Science, Framingham State University, Framingham, MA 01701, USA; eapostolidis@framingham.edu; 6 Department of Nutrition, University of Massachusetts, Amherst, MA 01003, USA

**Keywords:** chitosan-oligosaccharides, calorie restriction, adipocyte differentiations, 3T3-L1 cell, C/EBPβ, PPARγ, obesity

## Abstract

Excess body weight is a major risk factor for type 2 diabetes (T2D) and associated metabolic complications, and weight loss has been shown to improve glycemic control and decrease morbidity and mortality in T2D patients. Weight-loss strategies using dietary interventions produce a significant decrease in diabetes-related metabolic disturbance. We have previously reported that the supplementation of low molecular chitosan oligosaccharide (GO2KA1) significantly inhibited blood glucose levels in both animals and humans. However, the effect of GO2KA1 on obesity still remains unclear. The aim of the study was to evaluate the anti-obesity effect of GO2KA1 on lipid accumulation and adipogenic gene expression using 3T3-L1 adipocytes in vitro and plasma lipid profiles using a Sprague-Dawley (SD) rat model. Murine 3T3-L1 preadipocytes were stimulated to differentiate under the adipogenic stimulation in the presence and absence of varying concentrations of GO2KA1. Adipocyte differentiation was confirmed by Oil Red O staining of lipids and the expression of adipogenic gene expression. Compared to control group, the cells treated with GO2KA1 significantly decreased in intracellular lipid accumulation with concomitant decreases in the expression of key transcription factors, peroxisome proliferator-activated receptor gamma (PPARγ) and CCAAT/enhancer-binding protein alpha (CEBP/α). Consistently, the mRNA expression of downstream adipogenic target genes such as fatty acid binding protein 4 (FABP4), fatty acid synthase (FAS), were significantly lower in the GO2KA1-treated group than in the control group. In vivo, male SD rats were fed a high fat diet (HFD) for 6 weeks to induced obesity, followed by oral administration of GO2KA1 at 0.1 g/kg/body weight or vehicle control in HFD. We assessed body weight, food intake, plasma lipids, levels of alanine aminotransferase (ALT) and aspartate aminotransferase (AST) for liver function, and serum level of adiponectin, a marker for obesity-mediated metabolic syndrome. Compared to control group GO2KA1 significantly suppressed body weight gain (185.8 ± 8.8 g vs. 211.6 ± 20.1 g, *p* < 0.05) with no significant difference in food intake. The serum total cholesterol, triglyceride, and low-density lipoprotein (LDL) levels were significantly lower in the GO2KA1-treated group than in the control group, whereas the high-density lipoprotein (HDL) level was higher in the GO2KA1 group. The GO2KA1-treated group also showed a significant reduction in ALT and AST levels compared to the control. Moreover, serum adiponectin levels were significantly 1.5-folder higher than the control group. These in vivo and in vitro findings suggest that dietary supplementation of GO2KA1 may prevent diet-induced weight gain and the anti-obesity effect is mediated in part by inhibiting adipogenesis and increasing adiponectin level.

## 1. Introduction

Obesity, a major public health issue worldwide, is strongly associated with cardiometabolic complications including type 2 diabetes (T2D), non-alcoholic fatty liver disease (NAFLD), stroke, and cardiovascular disease [1,2,3,4]. Weight loss through lifestyle changes such as diet and exercise has been the first line of therapy and recommendation for T2D patients. In overweight and obese individuals with T2D, even modest loss of body weight (~5%) has been shown to not only improved glycemic control, but also significantly decrease cardiovascular events [5,6].

According to the world health organization (WHO), an estimated 38.2 million children under the age of 5 years were overweight or obese in 2019. In Africa, the number of overweight children under 5 has increased by nearly 24% percent since 2000. Almost half of the children under 5 who were overweight or obese in 2019 lived in Asia [6]. Therefore, preventing or managing obesity is becoming a public health priority. Although the exact etiology of obesity remains unclear, primary causes of obesity involves environmental and behavioral factors [7]. In humans and animals, obesity is characterized by increased fat mass, which is determined primarily by increased adipocyte number (hypertrophy) and/or adipocyte size (hypertrophy) [8]. Approximately 10% of adipocytes are renewed annually at all adult ages, however obese adults recruited more precursor cells (preadipocytes) and differentiated into new adipocytes than lean adults [9]. This was further supported by observations that showed increased recruitment and differentiation of adipogenic precursor cells in response to a long-term high fat diet in animals [10].

Adipogenesis is the process of the hyperplastic transformation of preadipocytes into adipocytes [11] with excess storage of lipids as triglycerides through increased lipogenesis leading to hypertrophic and dysfunctional adipocytes [12]. The cellular events and molecular processes of adipogenesis have been extensively studied using a murine 3T3-L1 preadipocyte cell line [13]. Adipogenesis and lipogenesis are initiated by the expression of differentiation-related transcription factors when the preadipocytes are exposed to adipogenic inducers [14,15]. Activation of key transcription factors such as PPARγ and C/EBPα in the early stage of adipogenesis upregulates the expression of multiple target genes responsible for adipocyte phenotype and lipid accumulation including fatty acid synthase (FAS), lipoprotein lipase (LPL), and fatty acid binding protein 4 (FABP), while lipolysis genes such as hormone sensitive lipase (HSL) are downregulated [15,16]. Recent evidence has demonstrated that dietary factors including several bioactive compounds can change not only the number of fat cells, but also fat cell size [17,18], thus serving as alternative agents for weight loss with potentially fewer side effects.

As a major regulator of lipid homeostasis, the liver is the organ most influenced by ectopic lipid accumulation. Thus, high fat diet-induced hepatic dyslipidemia is a common defect observed in obese individual [18]. The hallmark of dyslipidemia in obesity is hypertriglyceridemia due in part to increased free fatty acid (FFA) fluxes to the liver. This leads to increased plasma cholesterol, ALT (GPT, glutamate pyruvate transaminase), and AST(GOT, glutamate oxaloacetate transaminase) levels [19]. Moreover, numerous epidemiological studies have identified low adiponectin levels as an independent risk factor for NAFLD and liver failure [20]. Distinct from other adipokines serum levels of adiponectin are decreased in obesity and its related metabolic complications [21]. In addition, hepatic lipid accumulation has been shown to suppress the secretion of adiponectin from adipose tissue, which plays a key role in hepatic lipid oxidation and decreased lipogenesis [21]. Therefore, there has been increasing interest in identifying bioactive compounds that have potential for reducing triglyceride, non-HDL cholesterol, GPT and GOT and also enhancing adiponectin levels to help manage obesity and its associated metabolic diseases.

Chitosan oligosaccharides are water-soluble products obtained by the enzymatic digestion of chitosan and have shown various biological properties including anti-cancer, anti-inflammatory, and antioxidant activities [22,23,24,25]. Our previous studies have demonstrated that low molecular weight (<1000 Da) chitosan oligosaccharide (GO2KA1) exerts anti-diabetic effects by reducing hyperglycemia in both animal studies [25,26] and clinical trials [27,28]. Furthermore, GO2KA1 supplementation suppressed carbohydrate digesting enzymes and glucose absorption in an intestinal cell model [29]. We also found that supplementation of low molecular chitosan oligosaccharides significantly decreased absorption of dietary fat in the intestine and, the total plasma cholesterol level, but increased the serum adiponectin level [27]. While the beneficial effects of chitosan oligosaccharide in humans and animals are documented, the potential modes of action on lipid metabolism and the blood lipid profile are currently unclear. In our previous study, we showed that a low concentration of GO2KA1 (10 ug/mL) significantly enhanced adipocyte differentiation consistent with a marked increase in the expression of PPARγ and C/EBPα proteins and their downstream target genes, FABP4 and adiponectin [29]. However, GO2KA1 treatment at higher concentrations (50 and 100 µg/mL) did not further enhance the degree of differentiation and gene expression. In contrast to our findings, other reports showed that chitosan oligosaccharide treatment inhibited the differentiation of 3T3-L1 adipocytes as determined by Oil Red O staining of lipids and PPARγ mRNA expression [30]. The discrepancies are mainly due to the wide range of concentrations (0.5–4 mg/mL) and molecular weights of chitosan oligosaccharide (1–3 to 5–10 kDa) [30].

In the present study, the effects of higher concentrations of GO2KA1 (200 to 800 µg/mL) on the differentiation of 3T3-L1 adipocytes were investigated by measuring lipid accumulation and evaluating the expression levels of adipogenic transcription factors genes and their target genes. Further, we elucidated the potential in vivo effects of GO2KA1 on weight-management and the metabolic profile using a HFD-induced obesity model in Sprague-Dawley (SD) rats with and without oral administration of GO2KA1 for 3 weeks. We measured body weight, food intake, plasma glucose and lipids, levels of ALT (GPT) and AST (GOT) for liver function, and serum level of adiponectin, a marker for obesity and obesity-mediated metabolic syndrome.

## 2. Results

### 2.1. GO2KA1 Inhibits Adipocyte Differentiation

**Figure 1:**
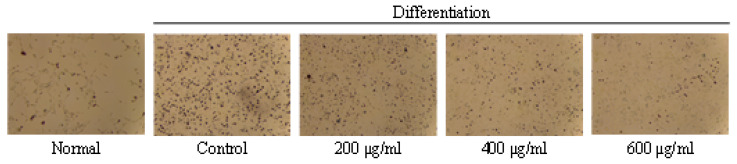

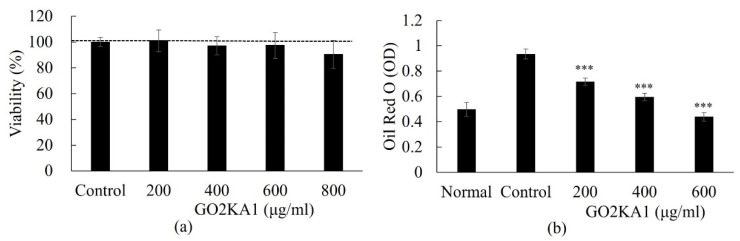
Effects of GO2KA1 on the viability (**a**) and lipid accumulation (**b**) of 3T3-L1 cells. (**a**): 3T3-L1 preadipocytes cells were seeded at a density of 1 × 10^4^ cells per well in a 96-well plates and incubated in culture medium at 37 °C for 24 h to allow attachment. The attached cells were either untreated control (CON) or treated with 200, 400, 600, or 800 µg/mL of GO2KA1 at 37 °C for 48 h. After 48 h of incubation. The effects of GO2KA1 on cell viability were measured by MTT assay. The data are presented as relative cell viability values. Data are the means ± standard deviation (S.D.) values of at least 3 independent experiments. (**b**): 3T3-L1 preadipocytes were grown and differentiated with the differentiation cocktail in the absence and presence of varying concentrations (0, 200, 400, and 600 μg/mL) of GO2KA1 throughout the differentiation for 8 days. After 8 days of differentiation, these cells were subjected to Oil Red O staining for Control and GO2KA1 to compare intracellular lipid accumulation (CON, control: without GO2KA1, normal: no differentiation). The results are expressed as the mean ± S.D (n ≥ 6). Significantly different from Control group (*** *p* < 0.001).

In our experiments, we investigated whether GO2KA1 at higher concentrations (200 to 800 µg/mL) inhibits adipodenesis during the differentiation of 3T3-L1 preadipocytes into mature adipocytes. To determine the cytotoxicity of GO2KA1 the effects of GO2KA1 on preadipocyte 3T3-L1 cell viability were determined using an established 3-(4,5-dimethylthiazol-2-yl)-2,5-diphenyltetrazolium bromide (MTT) assay. As shown in Figure 1a, when cells were treated with 200 to 800 µg/mL of GO2KA1 for 48 h at 37 °C, cytotoxicity was not observed at all concentrations compared to the control (without GO2KA1) cells (Figure 1a). Based on the result of the MTT assay, non-cytotoxic doses of GO2KA1 for anti-adipogenesis experiments were determined.

To understand the molecular basis of inhibitory effects of GO2KA1 on adipogenesis, we first attempted to clarify the expression levels of key transcription factors that are important for the potential anti-adipogenic effects of GO2KA1. The formation of lipid droplets in the adipocytes treated with 200 µg/mL of GO2KA1 were significantly blocked (*p* < 0.001) as confirmed by Oil Red O staining as shown in Figure 1b. Treatment with GO2KA1 during the differentiation process inhibited lipid accumulation in a dose-dependent manner (200, 400, and 600 µg/mL), as shown in Figure 1b.

### 2.2. GO2KA1 Decreases the Expression of Adipocyte Differentiation Related Genes

**Figure 2:**
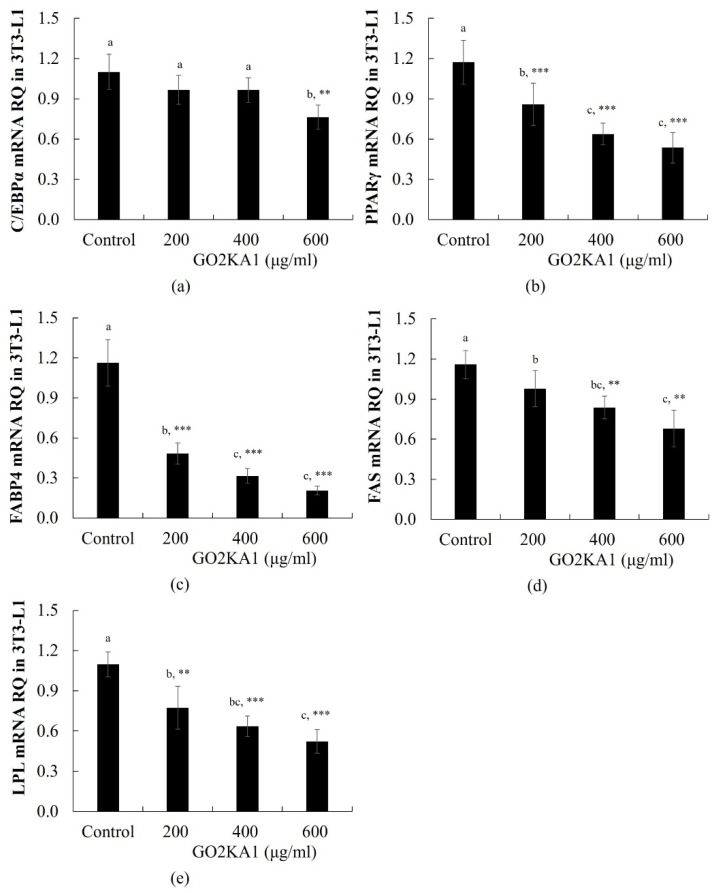
RT-real time PCR quantitative analysis of adipocyte differentiation related genes expression in the 3T3-L1. For real time PCR, we used SYBR green mix with gene-specific primers ((**a**): CEBP/α, (**b**): PPARγ, (**c**): FABP4, (**d**): fatty acid synthase (FAS), (**e**): lipoprotein lipase (LPL)). Each value is expressed as mean ± S.D. and is representative of at least three separate experiments. Different letters indicate statistically significant differences between groups with one-way ANOVA followed by Duncan’s test of *p* < 0.05. The results are expressed as the mean ± S.D (n ≥ 6). Statistical significances from control group were determined by Student’s *t*-test (** *p* < 0.01, *** *p* < 0.001).

Adipocyte differentiation is accompanied by the increased expression of several transcription regulators essential for terminal adipocyte differentiation such as PPARγ and C/EBPα [13]. PPARγ mRNA expression was significantly decreased following treatment with GO2KA1 at 200, 400, and 600 µg/mL, whereas C/EBPα mRNA level was significantly decreased only at 600 µg/mL GO2KA1 (Figure 2a,b). We further investigated whether the GO2KA1-induced PPARγ and C/EBPα regulation correlates with the expression of their target lipogenic genes, including fatty acid binding protein 4 (FABP4), fatty acid synthase (FAS), and lipoprotein lipase (LPL). Treatment with 200 or 600 µg/mL GO2KA1 markedly decreased the expression levels of FABP4, FAS, and LPL (Figure 2c–e).

### 2.3. GO2KA1 Alleviates HFD Induced Obesity in In Vivo Model

**Figure 3:**
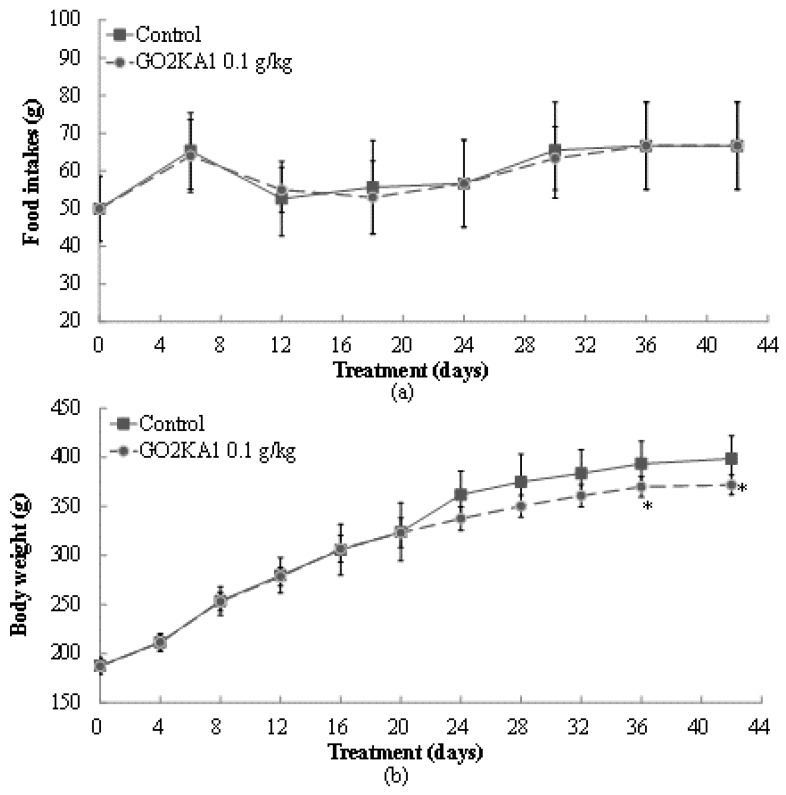
Changes in food intake (**a**) and body weight gains (**b**) before/after administration of GO2KA1. Male SD rats were free access to a high fat diet (HFD) (30% fat) to induce the weight gain for 42 days. During a HFD administration for 42 days, GO2KA1 was orally administrated (0.1 g/kg-body weight/day, peroral zonde injection) to Group II SD rats, 2 times per day (9–10 a.m. and 4–5 p.m.) with 0.05 g/kg-body weight each. Each point represents mean ± S.D. (n = 10). Food intake and body weight levels were compared between control (Group I) and treatment group (Group II) at each time point by unpaired Student’s *t*-test (* *p* < 0.05).

The effects of GO2KA1 administration (0.1 g/kg body weight) were evaluated in high fat diet (HFD)-induced obesity SD rat model for 42 days with dietary composition described in Table 1. After GO2KA1 administration with a HFD, we observed significant changes in food intake and weight gaining levels at day 24, 30, 36, and 42 day compared to control group (Figure 3). At the end day of the experiment, food consumption levels in control and GO2KA1 treatment group had no significant difference (Figure 3a). As seen in Figure 3b, GO2KA1 administration suppressed weight gain significantly compared to the control, even though there was no difference in food intake between treatment and control group (Figure 3b).

Initial weight of SD rats was 187 ± 8.13 g (control) and 187 ± 7.84 g (GO2KA1), and final weight of control and GO2KA1 treatment group was 398.7 ± 23.4 g and 372.0 ± 10.1 g, respectively (*p* < 0.05). Considering the changes from initial weight to final weight, GO2KA1 administration suppressed weight gain effectively around 35% compared to control group.

**Figure 4:**
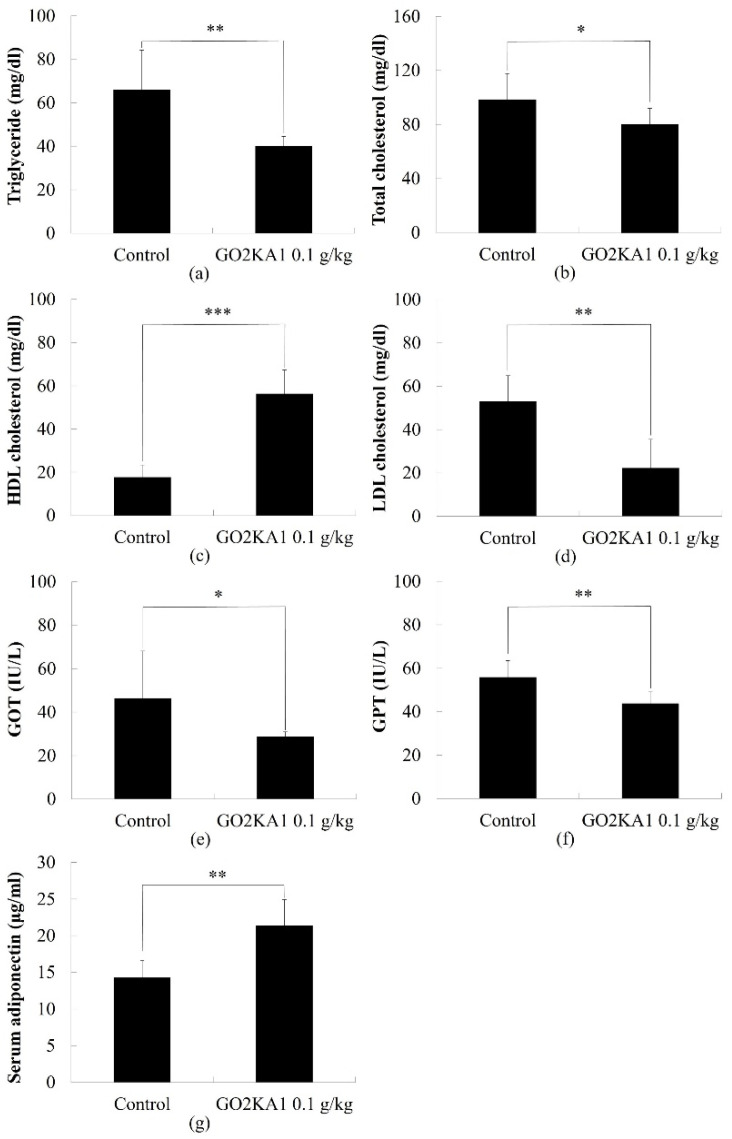
Comparison of Triglyceride (**a**), total cholesterol (**b**), HDL (**c**), LDL (**d**), GOT (**e**), GPT (**f**) and serum adiponectin (**g**) contents with or without administration of GO2KA1. Each point represents mean ± S.D. (n = 10). Triglyceride, total cholesterol, HDL, LDL, GOT, GPT, and serum adiponectin contents were compared between control (Group I) and treatment group (Group II) at each time point by unpaired Student’s *t*-test (* *p* < 0.05; ** *p* < 0.01; and *** *p* < 0.001).

To determine the effect of GO2KA1 on the plasma lipid profile, we evaluated the total cholesterol, LDL and HDL, and triglyceride. Aspartate aminotransferase (AST/GOT) and alanine aminotransferase (ALT/GPT) contents in blood. Compared to the control group, the total cholesterol level was decreased significantly after administration of GO2KA1 (Figure 4). Total cholesterol level in control and GO2KA1 administration group was 98.2 ± 19.4 mg/dL and 79.2 ± 12.5 mg/dL, respectively.

We found different cholesterol compositions between the two groups. GO2KA1 administration group significantly increased HDL levels, whereas LDL levels were significantly decreased compared to control group (Figure 4c,d).

We also observed the level of GOT and GPT contents in blood at the end day of experiment. We found that both GOT and GPT levels in the blood were significantly decreased in the GO2KA1 administration group. GOT contents in control and GO2KA1 administration group were 46.4 ± 21.7 IU/L, 28.7 ± 2.1 IU/L respectively, and GPT contents in the control and GO2KA1 administration groups were 55.9 ± 7.6 IU/L, GO2KA1: 43.8 ± 5.2 IU/L, respectively (Figure 4e,f).

Moreover, rat serum adiponectin levels after 42 days administration of GO2KA1 were determined and are displayed in Figure 4g. Compared with the control group, significant increases of serum adiponectin were shown in the GO2KA1 intake group (Figure 4g).

## 3. Discussion

In the present study, we demonstrated the anti-obesity effects of GO2KA1 in a rodent preadipocyte cell line, 3T3-L1 cells and also in an SD rat model of HFD-induced obesity. We found that treatment with GO2KA1 dose-dependently inhibited lipid accumulation and the differentiation of 3T3-L1 preadipocytes into adipocytes. Treatment with GO2KA1 also decreased the expression of C/EBPα and PPARγ, key transcription factors that regulate adipogenesis and fat metabolism. Moreover, GO2KA1 treatment inhibited their downstream target genes including FABP4, LPL, and FAS that are adipocyte-specific and are also involved in maintaining the adipocyte phenotype. The cytotoxicity assay showed that GO2KA1 was not toxic to 3T3-L1 cells at the concentrations used in this study.

We also evaluated the anti-obesity effect of GO2KA1 administration in an SD rat model of HFD-induced obesity for 42 days and this was compared to the control. We observed that the GO2KA1 treated group showed significantly decreased body weight compared to the control without a significant difference in food intake. Further, GO2KA1 administration suppressed the triglyceride level and showed a positive plasma cholesterol profile by increasing the HDL level and decreasing the LDL level. To elucidate the mode of action of GO2KA1 in fat metabolism, we evaluated GOT, GPT, and adiponectin in serum. GOT and GPT were significantly decreased, and adiponectin expression was significantly increased compared to the control. The above findings suggest that GO2KA1 prevent the progression of obesity with beneficial effects.

In our previous study, we showed that GO2KA1 supplementation in *db/db* mice exerted significant inhibitory effects on mRNA expression of intestinal carbohydrate-digesting enzymes in the small intestine, and decreased body weight [26]. In addition, we found in our previous clinical studies that GO2KA1 supplementation significantly lowered fasting glucose levels and decreased the size of subjects’ waists after 12 weeks compared to placebo subjects [27,28]. These data from the animal and clinical studies suggest that GO2KA1 supplementation markedly retarded glucose digestion and absorption after sugar administration, and is consistent with a line of evidence that the chitosan oligosaccharide plays an important role in glucose homeostasis. Intestinal glucose transporters and carbohydrate digesting enzymes expressed in the apical membrane play a critical role in glucose absorption. Deletion of glucose transporters and inhibition of carbohydrate enzymes failed to transport glucose from the intestine to the body.

Epidemiological studies have shown a positive relationship between dietary fat intake and obesity [16]. While the cause of obesity is complex, numerous studies have shown that a diet high in fat is clearly a contributing factor for weight gain and the global prevalence of obesity. Obesity is characterized by hypertrophic adipocytes and adipose tissue dysfunction, which limit the storage of triglycerides from dietary fatty acids. Reduced uptake of dietary fatty acids by adipose tissue leads to hypertriglyceridemia and reduced HDL cholesterol levels in the blood. A strong positive correlation was demonstrated between body fat and serum-cholesterol and serum-triglyceride levels. Therefore, we evaluated body weight as well as triglyceride and cholesterol in blood. Our data suggest that GO2KA1 administration significantly decreased body weight, triglyceride and total cholesterol levels whereas, HDL cholesterol level significantly increased. Our findings suggest that GO2KA1 has the potential to control weight gain and this is possibly through its inhibitory effect on adipogenesis.

Numerous of adipocyte-derived secretory factors have been identified as playing a role in the maintenance of lipid homeostasis in the liver and a critical mediator is adiponectin [31,32]. Adiponectin is secreted from adipose tissue into the blood stream and attaches to adiponectin receptors (adipoR1 and adipoR2) in the liver. Adiponectin has been shown to enhance insulin sensitivity and maintains a healthy liver by decreasing lipogenesis and increasing β-oxidation through activation of AMP protein kinase [33,34]. Although adiponectin is secreted from adipose tissue, adiponectin secretion is decreased in the obese state which leads to lipid accumulation including triglyceride and non-HDL cholesterol. Therefore, enhanced serum adiponectin levels have been emphasized in preventing obesity. A study showed that 8-week administration of chitosan oligosaccharides increased serum adiponectin in obese rats. We also observed this in an SD rat model which shown in Figure 4. These results support our previous clinical studies [28] with pre-diabetic subjects that long-term GO2KA1 consumption enhance the serum adiponectin level, and this could be a possible mechanism for weight management.

GOT/GPT is located in the liver more than in any other organs. The normal range for GOT is 8–40 IU/mL, and GPT is 5–30 IU/ ml. The high fat diet itself induces a significant increase in both GOT and GPT levels which induces obesity and a fatty liver.

Our findings suggest that administration of the chitosan oligosaccharide (GO2KA1) can help reduce body-fat accumulation in an SD rat model. Further studies are required to evaluate the potential anti-obesity effect of the chitosan oligosaccharide (GO2KA1) in humans. The identification of the cellular mechanism of GO2KA1 in view of hepatic lipogenesis and fatty acid oxidation using an in vitro model, provides great potential to identify novel targets for the prevention of obesity. In addition, it is necessary to evaluate glucose and lipid metabolism in different tissues including skeletal muscle, and the liver. The use of different preclinical animal models is needed to support the mode of action of GO2KA1.

## 4. Materials and Methods

### 4.1. Materials

Chitosan oligosaccharides classified by molecular weight (GO2KA1; MW < 1000 Da) were purchased from Kunpoong Bio Co. Ltd. (Jeju, Korea). Corn starch, casein, vitamin mix, mineral mix, calcium phosphate and sodium chloride were purchased from Raon Bio (Yonginsi, Korea). Total cholesterol and total glyceride kits were purchased from Stanbio laboratory (Boerne, TX, USA). Unless noted, otherwise, all chemicals were purchased from Sigma-Aldrich Co. (St. Louis, MO, USA). The fast SYBR real-time PCR master mix, Dulbecco’s modified Eagle’s medium (DMEM), fetal bovine serum (FBS), bovine calf newborn serum (BCS), penicillin-streptomycin (P/S), and trypsin-EDTA were obtained from Life Technologies (Grand Island, NY, USA). Adiponectin ELISA kit was purchased from Thermo Fisher Scientific (Invitrogen, Carlsbad, CA, USA). 3T3-L1 cells (ATCCV^®^CL-173^TM^) were used below passage 12. 3T3-L1 preadipocytes were propagated and cultured in DMEM medium supplemented with 10% BCS and 1% P/S until confluent and maintained for additional 2 days and differentiated as reported previously [26] with or without GO2KA1.

### 4.2. Determination of Cell Viability

The effects of GO2KA1 on 3T3-L1 cell viability were determined using an established MTT assay. Briefly, the 3T3-L1 preadipocytes cells were seeded at a density of 1 × 10^4^ cells per well in a 96-well plates and incubated in culture medium at 37 °C for 24 h to allow attachment. The attached cells were either untreated control (CON) or treated with 200, 400, 600, or 800 µg/mL of GO2KA1 at 37 °C for 48 h. After 48 h of incubation the cells were washed with phosphate-buffered saline (PBS) prior to the addition of MTT (0.5 µg/mL PBS) and incubated at 37 °C for 2 h. Formazan crystals were dissolved with dimethyl sulfoxide (100 µl/well) and detected at OD_570_ with a model Emax (Molecular Devices, Sunnyvale, CA, USA).

### 4.3. Oil Red O (ORO) Staining

To determine the degree of differentiation as measured by intracellular lipid content, ORO was performed as previously described [29]. Briefly, 3T3-L1 preadipocytes were cultured in DMEM/high-glucose medium containing 10% calf serum until confluent (Day −2) and maintained for an additional 2 days (until Day 0). Differentiation was induced on Day 0 by the addition of 0.5 mmol/L methylisobutylxanthine, 1 μmol/L dexamethasone, 1.0 μg/mL insulin and 10% fetal bovine serum (FBS) in DMEM. After 48 h (Day 2), the medium was replaced with DMEM containing 1.0 μg/mL insulin and 10% FBS. Medium was changed every 2 days thereafter until the cells were collected for analysis [29]. GO2KA1 was reconstituted as 1000 µg/mL stock solutions in DMSO (dimethyl sulfoxide) and added at the indicated concentrations on Day 0. Cells were cultured with GO2KA1 until cells were collected for analysis. After 8 days of differentiation 3T3-L1 adipocytes were washed with 4% paraformaldehyde once and fixed with 4% paraformaldehyde for 20 min at room temperature. Cells were then washed with 60% isopropanol once and stained with diluted Oil Red O solution for 30 min. After photographing the stained cells, the dye retained in 3T3-L1 cells was eluted with 100% isopropanol and the absorbance was measured by a microplate reader (SpectraMax M2, Molecular Devices, Sunnyvale, CA) at 490 nm.

### 4.4. Quantitative Real-Time PCR

Quantitative reverse transcription-polymerase chain reaction (qRT-PCR). Total RNA was isolated with TRIzol^®^ plus RNA purification kit according to manufacturer’s protocol (Life Technologies, Grand Island, NY, USA). One microgram of total RNA was used to synthesize cDNA using Revert Aid First Strand cDNA Synthesis kit (Thermo Scientific, Waltham, MA, USA). The reaction was performed with Fast SYBR^®^ Green Master Mix containing 1 µM of primer pair and 100 ng of cDNA under 40 cycles with each of 95 °C for 1 sec and 58 °C for 20 s. Relative levels of the target mRNA expression were determined by ViiA^TM^ 7 real-time PCR system (Life technologies, Grand Island, NY, USA), normalized to GAPDH calculated with the 2^−(ΔΔ^nd^)^ method. The primer sequences are listed in Table 2. All the results were normalized to the housekeeping gene, glyceraldehyde 3-phosphate dehydrogenase (GAPDH), to control for variations in mRNA concentrations. Relative quantification was performed using the comparative delta-delta Ct method according to the manufacturer’s instructions (Applied Biosystems).

### 4.5. In Vivo Experimental Design

Five-week-old male Sprague-Dawley (SD) rats were purchased from Joongang Experimental Animal Co. (Seoul, Korea) and fed a high fat diet (30% fat) (Table 3) for 42 days. After 3 days the normal diet (Pico 5053) was switched to a high fat-diet (HFD) (Oriental Bio. Co., Seongnam, Korea) for 42 days. During the HFD administration for 6 weeks, rats were divided into two groups, one group received HFD and the second group received HFD with GO2KA1. GO2KA1 was orally administrated, 2 times a day (9–10 a.m. and 4–5 p.m.). Distilled water (D.W.) was used as a vehicle for a solution of the experimental compound, GO2KA1. In a control group, D.W. was used as a vehicle for oral administration without GO2KA1 using a zonde injection needle. The dose of each GO2KA1 administration was 0.05 g/kg-body weight, yielding a final dose of 0.1 g/kg-body weight/day. The animals were housed in individual cages in a room with a 12 h light/dark cycle (lights on from 06:00 h) with 50% ± 7% relative humidity. In this study, ten SD rats were used for each group. The experimental protocols were approved by the Institutional Animal Care and Use Committee (IACUC) of the Hannam University (Approval number: HNU2014-0019). The rats had free access to water throughout the experimental period. The rats were anesthetized with pentobarbital and sacrificed, and blood was collected and serum was processed and stored at −80 °C until used.

Group I: Control.

Group II: GO2KA1 0.1 g/kg-body weight/day.

### 4.6. Blood Analysis

The plasma total cholesterol and total glyceride concentration was measured using a kit (Stanbio lab., Boerne, TX, USA). Serum adiponectin levels in SD rats were detected by ELISA kit (Invitrogen, Carlsbad, CA, USA).

### 4.7. Statistical Analysis

Statistical analyses were carried out using the statistical package SPSS 10 (Statistical Package for Social Science, SPSS Inc., Chicago, IL, USA) program and significance of each group was verified with the analysis of One-way analysis of variance (ANOVA) followed by the Duncan’s multiple range test of *p* < 0.05 and the Student’s *t*-test for comparison of means.

## Figures and Tables

**Table 1:** Effects of GO2KA1 treatment on various parameters in SD rats.

	Control	GO2KA1
Initial body weight (g)	187.8 ± 8.13	187.2 ± 7.84
Body weight (g)	398.7 ± 23.4	372.0 ± 10.1 *
Body weight gaining (g)	211.67 ± 20.18	185.89 ± 8.89 *
Triglyceride (mg/dL)	66.0 ± 18.2	40.0 ± 4.6 **
Total Cholesterol (mg/dL)	98.2 ± 19.4	79.2 ± 12.5 *
HDL (mg/dL)	17.6 ± 5.7	56.3 ± 10.9 ***
LDL (mg/dL)	53.1 ± 11.7	22.3 ± 13.1 ***
AST (GOT, IU/L)	46.4 ± 21.7	28.7 ± 2.1 *
ALT (GPT, IU/L)	55.9 ± 7.6	43.8 ± 5.2 **
Adiponectin (μg/mL)	14.3 ± 2.3	21.4 ± 3.5 **

Each experiment was compared between control (Group I) and GO2KA1 administration group (Group II) at each time point by unpaired Student’s *t*-test (* *p* < 0.05; ** *p* < 0.01; and *** *p* < 0.001).

**Table 2:** Primer for real-time quantitative PCR.

Genes	Primer Sequences	
Accession Number	Forward (5′-3′)	Reverse (5′-3′)
GAPDH	CGTCCCGTAGACAAAATGGT	TTGATGGCAACAATCTCCAC
NM_008084		
PPARγ	GAAAGACAACGGACAAATCACC	GGGGGTGATATGTTTGAACTTG
NM_011146		
C/EBPα	TTGTTTGGCTTTATCTCGGC	CCAAGAAGTCGGTGGACAAG
NM_007678		
FABP4	AGCCTTTCTCACCTGGAAGA	TTGTGGCAAAGCCCATC
NM_024406		
FAS	TGATGTGGAACACAGCAAGG	GGCTGTGGTGACTCTTAGTGATAA
NM_007988		
LPL	GGACGGTAACGGGAATGTATGA	TGACATTGGAGTCAGGTTCTCTCT
NM_008509		

PCR, polymerase chain reaction; PPARγ, peroxisome proliferator-activated receptor γ; C/EBPα, CCAAT/enhancer-binding protein α; FAS, fatty acid synthase; LPL, lipoprotein lipase; GAPDH, glyceraldehyde 3-phosphate dehydrogenase.

**Table 3:** Composition of high fat-diet (g/kg).

High Fat Diets (g/kg)	
Corn starch	321
Sucrose	100
Casein	200
Corn oil	100
Lard	200
Cellulose	30
DL-methionine	2
Vitamin mix ^(1)^	10
Mineral mix ^(2)^	35
Choline bitartrate	2

^(1)^ Vitamin mixture: AIN-93VX; ^(2)^ Mineral mixture: AIN-93G.

## Data Availability

The data will be available on request.
